# Contrast-to-noise ratio comparison between X-ray fluorescence emission tomography and computed tomography

**DOI:** 10.1117/1.JMI.11.S1.S12808

**Published:** 2024-10-15

**Authors:** Hadley DeBrosse, Giavanna Jadick, Ling Jian Meng, Patrick La Rivière

**Affiliations:** a University of Chicago, Department of Radiology, Chicago, Illinois, United States; b University of Illinois Urbana-Champaign, Department of Nuclear, Plasma, and Radiological Engineering, Urbana, Illinois, United States

**Keywords:** X-ray fluorescence emission tomography, computed tomography, contrast-to-noise ratio, X-ray fluorescence computed tomography, detection limit

## Abstract

**Purpose:**

We provide a comparison of X-ray fluorescence emission tomography (XFET) and computed tomography (CT) for detecting low concentrations of gold nanoparticles (GNPs) in soft tissue and characterize the conditions under which XFET outperforms energy-integrating CT (EICT) and photon-counting CT (PCCT).

**Approach:**

We compared dose-matched Monte Carlo XFET simulations and analytical fan-beam EICT and PCCT simulations. Each modality was used to image a numerical mouse phantom and contrast-depth phantom containing GNPs ranging from 0.05% to 4% by weight in soft tissue. Contrast-to-noise ratios (CNRs) of gold regions were compared among the three modalities, and XFET’s detection limit was quantified based on the Rose criterion. A partial field-of-view (FOV) image was acquired for the phantom region containing 0.05% GNPs.

**Results:**

For the mouse phantom, XFET produced superior CNR values (CNRs=24.5, 21.6, and 3.4) compared with CT images obtained with both energy-integrating (CNR=4.4, 4.6, and 1.5) and photon-counting (CNR=6.5, 7.7, and 2.0) detection systems. More generally, XFET outperformed CT for superficial imaging depths (<28.75  mm) for gold concentrations at and above 0.5%. XFET’s surface detection limit was quantified as 0.44% for an average phantom dose of 16 mGy compatible with *in vivo* imaging. XFET’s ability to image partial FOVs was demonstrated, and 0.05% gold was easily detected with an estimated dose of ∼81.6  cGy to a localized region of interest.

**Conclusions:**

We demonstrate a proof of XFET’s benefit for imaging low concentrations of gold at superficial depths and the feasibility of XFET for *in vivo* metal mapping in preclinical imaging tasks.

## 1 Introduction

X-ray fluorescence emission tomography (XFET) is an emerging imaging modality designed to map the spatial distribution of exogenous metals. XFET relies on a novel imaging geometry contingent on photon-counting detection. XFET is similar to X-ray fluorescence computed tomography (XFCT) in that it is a functional imaging modality designed to image very low concentrations of metal. Similar to XFCT, XFET utilizes a pencil beam of incident X-rays to cause photoelectric interactions with metals and subsequent emission of characteristic, or fluorescence, X-rays. Yet, XFET has several differences and potential advantages over conventional XFCT. Most notably, XFET utilizes slit or pinhole apertures coupled to spatial- and energy-resolving detectors. Most recently, benchtop XFET has utilized high-energy X-ray imaging technology (HEXITEC) cadmium telluride (CdTe) detectors.^1^ These detectors count and assign energies to detected fluorescence photons with an improved full energy spectrum resolution of 1 keV at an energy relevant to this work.^2^ High spectral resolution is necessary for fluorescence imaging to separate the fluorescence signal from Compton scatter contamination; the 1-keV energy resolution also allows XFET to simultaneously distinguish among many different metals of interest, which have distinct fluorescence energies. These metals may be distinguishable with state-of-the-art photon-counting CT (PCCT) but may appear identical on a conventional, energy-integrating CT (EICT). This detector system, combined with the novel slit or pinhole apertures that characterize XFET, encodes spatial information about metals within an object. During XFET imaging, fluorescence X-rays induced from metals are counted and can be traced back to their point of emission with finite resolution determined by the slit width and detector pixel width. Therefore, XFET directly forms three-dimensional (3D) images of metal within objects, without the need for accumulating a sinogram or noise-amplifying tomographic image reconstruction.^3^^,^^4^ XFET’s critical modification of conventional XFCT—from a non-imaging detector to pixelated, photon-counting detectors coupled with slit apertures—allows for demonstrated sensitivity improvements.^1^^,^^3^ Furthermore, XFET’s mechanism of direct imaging allows for partial field-of-view (FOV) or region-of-interest (ROI) imaging, making it potentially more dose-efficient than conventional XFCT, which typically requires a nearly full sinogram acquisition to view the same region.^5^

XFET’s primary limitation is imaging depth. This limitation arises not only from the depth of its pencil beam penetration in tissue but also from the attenuation of the induced fluorescence X-rays. This limitation is also present and potentially more limiting in XFCT; due to its full sinogram requirement, the fluorescent signal may extinguish during certain portions of the object rotation and sinogram collection.^6^ Both XFCT and XFET are limited to applications in either preclinical studies or superficial clinical studies.

Fortunately, there are many preclinical and clinical studies that could benefit from XFET imaging as metals and metal-based drugs are increasingly being explored for their therapeutic potential. Among these metals, gold nanoparticles (GNPs) have particular promise due to their biocompatibility, functionalization, radiation enhancement, and photothermal properties.^7^^,^^8^ Previous preclinical studies have shown that GNPs, when injected into a tumor prior to radiation therapy treatment, can enhance the dose to the lesion on the order of 200% for certain gold concentrations and beam energies.^9^^,^^10^ As of 2022, a few ongoing clinical trials have used gold for photothermal ablation and similar therapies.^11^ Photothermal ablation employs a near-infrared laser to heat and ablate regions containing GNPs and is therefore limited in application to relatively superficial tumors for which the infrared penetration in tissue is not diminished.^11^^,^^12^ This depth limitation of photothermal ablation therapy is compatible with XFET imaging. Apart from radiation enhancement and photothermal ablation, GNPs and drugs incorporating gold have also shown success for targeted drug delivery *in vitro*, including in human breast and colon cancer cell lines.^13^^–^^15^ For shallow depths, XFET could map the spatial distribution of these metals, potentially decreasing clinical side effects or aiding in the research and development of these drugs in preclinical biodistribution experiments.

In advancing XFET toward preclinical and clinical imaging, we explored approaches for novel image formation, improved attenuation correction, and optimized detector placement. XFET does not require image reconstruction, but its mechanism of direct imaging allows for the joint estimation of both metal and attenuation maps from emission data alone. We previously developed a joint image reconstruction algorithm that estimates and corrects for attenuation and more accurately quantifies the metal in an object.^16^ We have also explored the conditioning of the inverse problem and reconstruction accuracy as a function of detector number and placement.^17^ Although both of these studies were crucial for XFET’s advancement toward preclinical imaging applications, it is still necessary to establish proof of benefit before XFET can be translated into these spaces. Specifically, a comparison with more conventional imaging modalities such as EICT or clinically emerging modalities such as PCCT would demonstrate XFET’s preclinical and clinical advantages.^18^

Previous studies have compared XFCT with computed tomography (CT) in the task of achieving high contrast in metal regions and, under some conditions, have shown that XFCT outperforms CT in this task. Some studies have used variations of XFCT that incorporate a cone beam with a flat-panel photon-counting detector and pinhole collimation. One group demonstrated the feasibility of such a geometry by imaging gadolinium (Gd) solutions with concentrations as low as 2  mg/mL^19^ and showed that their system outperformed CT in producing greater contrast-to-noise ratios (CNRs) of Gd inserts and achieving lower Gd detection limits.^20^ Another study that used a similar XFCT system to map GNPs *in vivo* demonstrated enhanced sensitivity and specificity of GNP detection compared with CT.^21^ Other groups have compared conventional XFCT with K-edge CT both analytically and in simulation.^22^^,^^23^ These studies have consistently found that XFCT produced greater CNRs than K-edge CT at GNP concentrations below 0.4% by weight.^22^^,^^23^ It remains to be seen how the sensitivity between CT and XFET compares under various gold concentrations and imaging conditions.

In this work, we compare two photon-counting detector modalities (XFET and PCCT) and one energy-integrating detector modality (EICT) to characterize the conditions under which XFET outperforms CT with either detection system. This work provides the first comparison of XFET, PCCT, and EICT. We use Monte Carlo and raytracing simulations to compare the three imaging modalities for two phantoms. A mouse whole-body (MOBY) digital phantom^24^ containing various gold concentrations in different tissues was used to demonstrate the feasibility of XFET in a preclinical context. Another numerical phantom was designed to test the effect of beam depth and gold concentration on CNR and to quantify the detection limit of XFET. XFET’s ability to image partial FOVs is also demonstrated. The results of this study provide the depth and sensitivity limits of XFET compared with other photon-counting and conventional imaging modalities and guide discussion about potential clinical and preclinical applications of XFET.

## 2 Methods

We compared the performance of simulated XFET and CT in the task of detecting regions of low concentrations of gold nanoparticles. Two phantoms were used: (1) a realistic digital mouse phantom (MOBY) containing gold in the kidneys, a hind leg tumor, and various other organs and (2) a cylindrical soft tissue phantom containing spheres of varying gold concentrations. XFET and CT simulations were designed to be approximately dose-matched and comparable in physical scale, as described in Sec. 2.3.

### 2.1 XFET Scanner Design and Monte Carlo Parameters

**Fig. 1:**
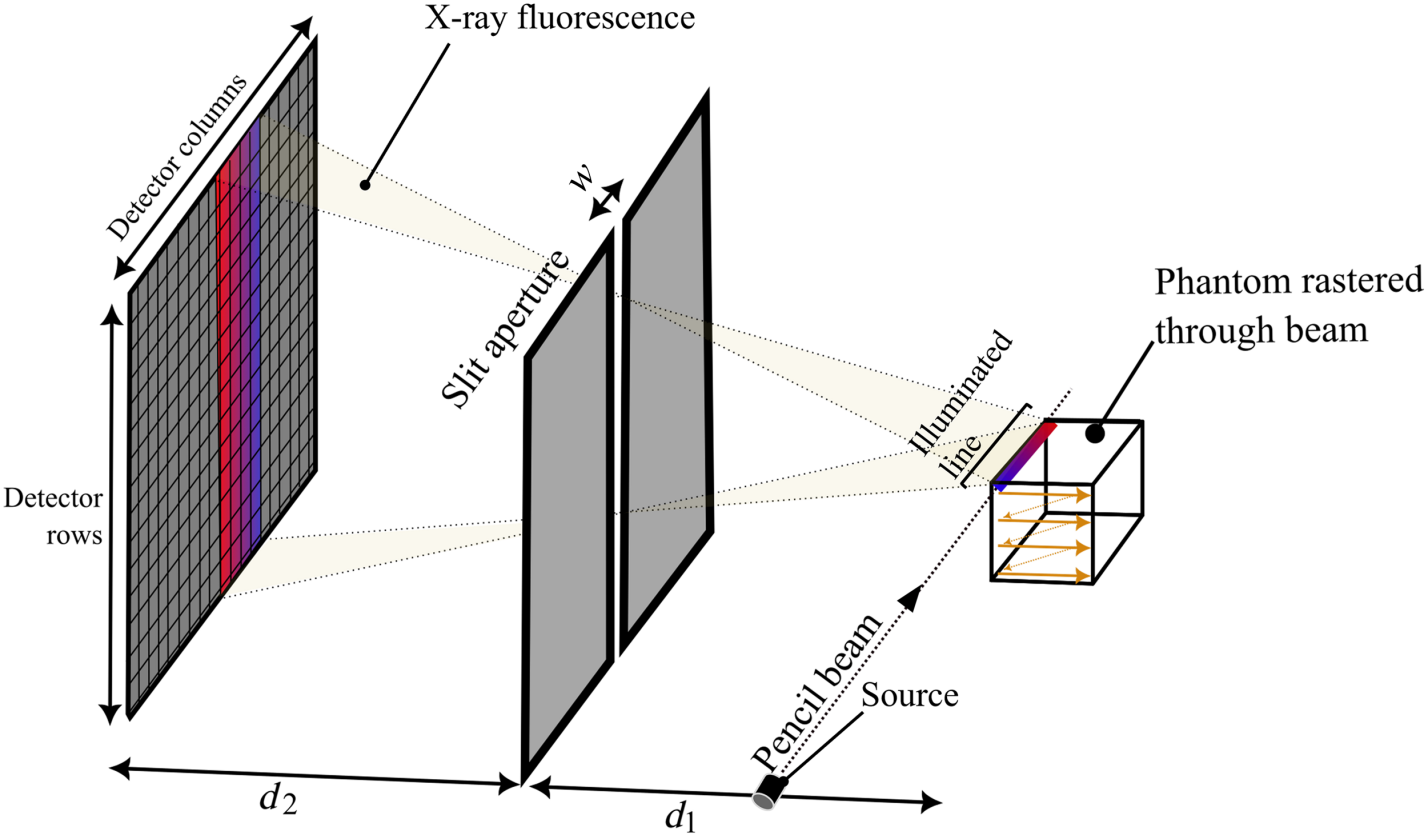
Mechanism of XFET imaging. The phantom is rastered through the 120-kVp X-ray pencil beam, resulting in fluorescent emissions along a line of illumination. Fluorescent emissions are directly mapped to the detector plane, spatially inverted, which is demonstrated by the red and blue regions in both the object and detector planes (not to scale).

Figure 1 displays the XFET’s mechanism of direct imaging. A collimated X-ray source illuminates a line within the phantom. Provided that the X-ray pencil beam contains X-rays with energies above the K-edge of the metal of interest, metal atoms along the line of illumination interact with those X-rays primarily through the photoelectric effect and subsequently emit X-ray fluorescence. X-ray fluorescence photons that are emitted from a point on the line are directly detected by the pixelated, energy-sensitive detector plane after passing through a slit aperture of width w. As the phantom is rastered through the stationary pencil beam, a metal map is formed directly without the need for tomographic image reconstruction.

**Fig. 2:**
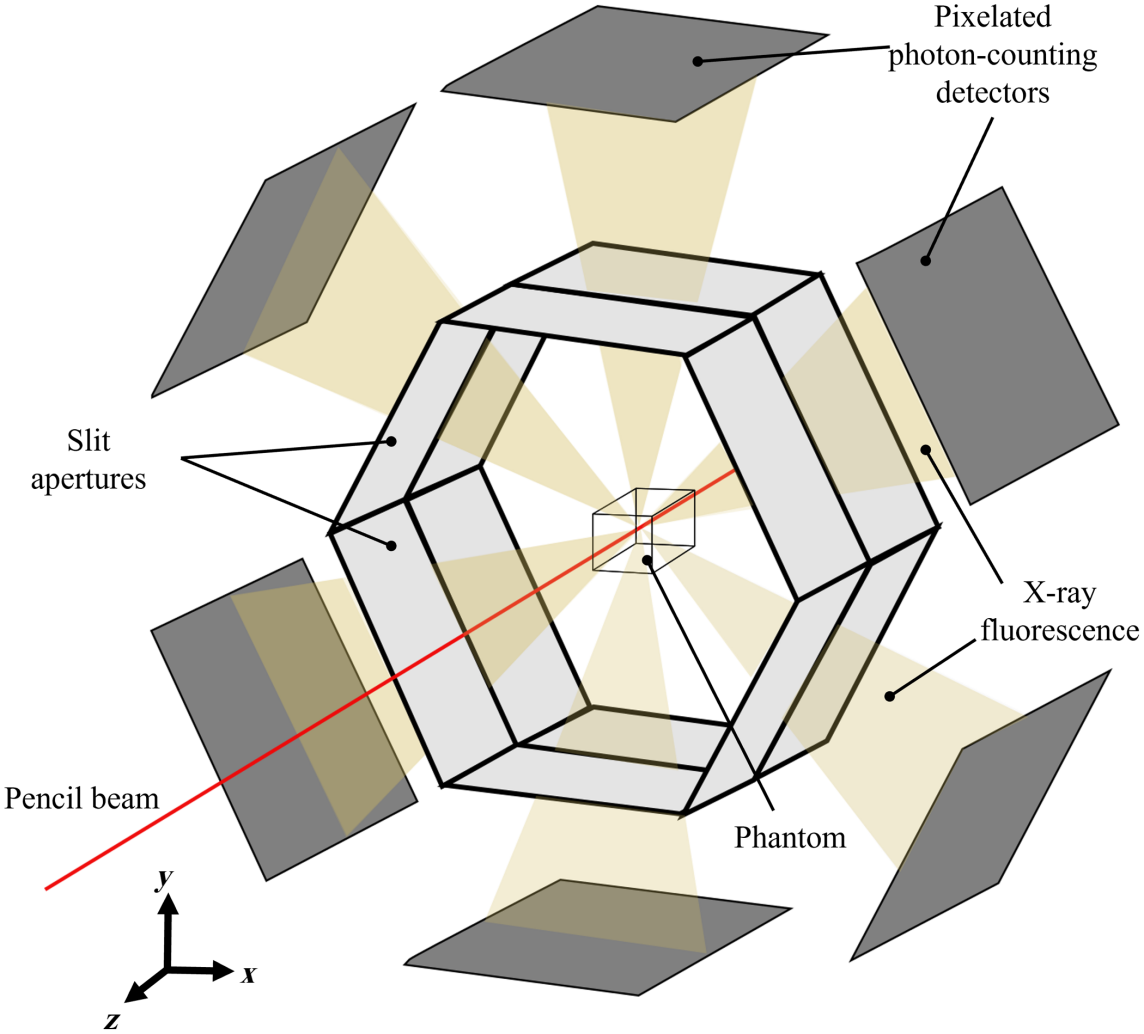
Illustration of XFET imaging geometry used in this work, with six hexagonal slit–detector combinations (not to scale).

Despite the simplified XFET diagram in Fig. 1, the XFET imaging system used in this work was approximately modeled after our benchtop system, which uses a full-ring detector geometry.^1^ This XFET geometry, shown in Fig. 2, positions the X-ray pencil beam perpendicularly to six hexagonally arranged lead (Pb) slit apertures sitting in front of the detectors.

All XFET simulations in this study were performed with TOPAS, a Geant4 wrapper Monte Carlo software,^25^ using a physics package suitable for medical applications, g4em-standard_opt3. Phantoms placed at the isocenter were rastered horizontally and vertically through a two-dimensional 120-kVp polychromatic pencil beam with no angular divergence. Each phantom was rastered through the beam at horizontal and vertical increments of choice; this resolution choice varied with computational allowances for each phantom as specified below. To decrease the computation time, only the portion of the spectrum above 65.263 keV was simulated. Because only energies above the K-edge of gold will contribute to fluorescence production, using this limited spectrum still scored all relevant photons for image formation, including gold $K_{\alpha_{1}}$ fluorescent emissions at 68.8 keV as well as scatter in the neighboring energy bins. This reduced the computational burden significantly by simulating only ∼15% of the counts that would otherwise be used with the full spectrum model.

Lead apertures were placed at a distance $d_{1}=83\;\mathrm{mm}$ from the isocenter in a hexagonal pattern, as shown in Fig. 2. Apertures were 1-mm-thick, which allowed for only ∼1% photon penetration at the fluorescent energy of interest and contained slits of width w=0.5  mm positioned at z=0  mm.

Each lead aperture was placed between the isocenter and a detector at a distance $d_{1}+d_{2}=166\;\mathrm{mm}$ from the isocenter. There were six detector planes also arranged hexagonally in a full-ring geometry. Each detector plane had dimensions 92  mm×120  mm and was divided into 46×120 spatial bins (pixels), such that the width of each detector column was 1 mm and each row was 2 mm. Each aperture plane was equidistant from its corresponding detector and the isocenter ($d_{1}=d_{2}$). This geometry, coupled with the slit width of 0.5 mm, allowed for the 1:1 mapping of detector pixel z-width to the 1 mm axial resolution of the object. The spectral information offered by XFET’s detectors allowed for scoring of X-ray fluorescence and scattered photons in 1-keV energy bins. We used 100% counting efficiency in XFET simulations for a standardized comparison to idealized CT, as discussed further in Sec. 2.2.

**Fig. 3:**
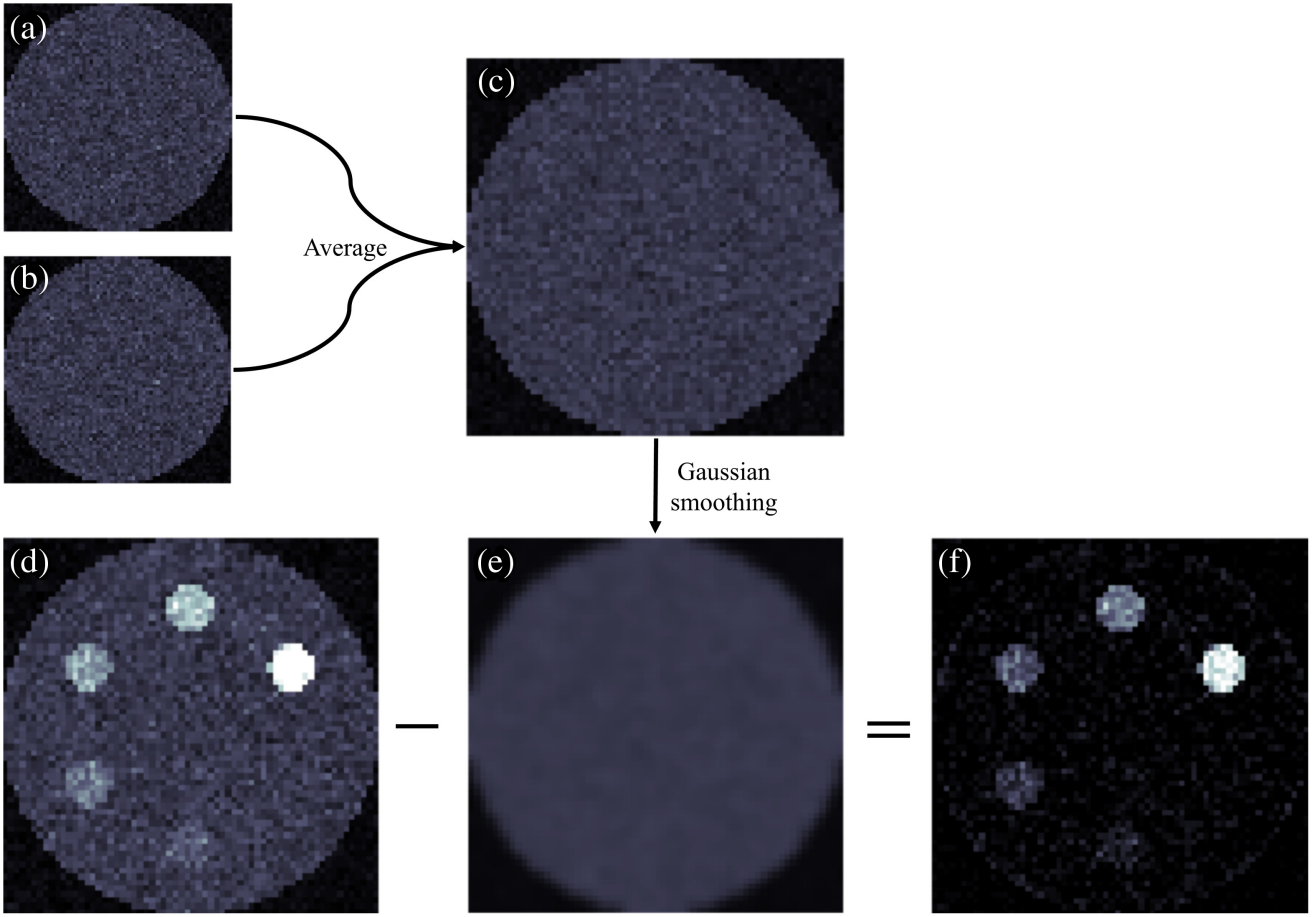
Diagram illustrating the method of Compton scatter estimation and correction. (a) One image slice displaying the sum of detector counts for the 67- to 68-keV energy bin. (b) One image slice displaying the sum of detector counts for the 69- to 70-keV energy bin. (c) Average of panels (a) and (b) representing the Compton scatter background. (d) One image slice displaying the sum of detector counts for the 68- to 69-keV energy bin, corresponding to the $K_{\alpha_{1}}$ fluorescent energy of gold. (e) Compton scatter background (c) smoothed with Gaussian kernel. (f) Difference of panels (d) and (e): the final image after Compton background subtraction.

Images were formed by summing photon counts in the appropriate energy bin across all detector rows for each object voxel. Because XFET is a direct imaging modality, no image reconstruction was needed or applied. Due to the nature of this comparison study, no attenuation correction measures were taken. This allowed us to find the depth threshold at which CT outperforms XFET due to the attenuation of XFET’s primary pencil beam. The XFET axial resolution, originally 1 mm, was rebinned to 2 mm for increased contrast with some loss of spatial resolution. The spectral information of the detected counts was used for Compton scatter background subtraction. The energy bin containing gold $K_{\alpha_{1}}$ fluorescence counts also contains contamination from Compton scatter at the same energy. Therefore, the Compton scatter background was removed by first finding the sum of detected counts in the two adjacent energy bins to the fluorescent energy. These counts were averaged to find the Compton scatter background image, smoothed across the object plane with a Gaussian kernel with σ=1 pixel and then subtracted from the image to reveal an image consisting of, in principle, only fluorescence photons. This method of scatter subtraction is illustrated in Fig. 3.

### 2.2 CT Scanner Design

Fan-beam CT simulations were completed in Python using Siddon’s raytracing algorithm,^26^ with fan-beam filtered backprojection (FFBP) reconstruction. CT simulations were designed to be approximately dose- and scale-matched to XFET simulations; both modalities are well-suited for imaging mouse-sized objects.

**Fig. 4:**
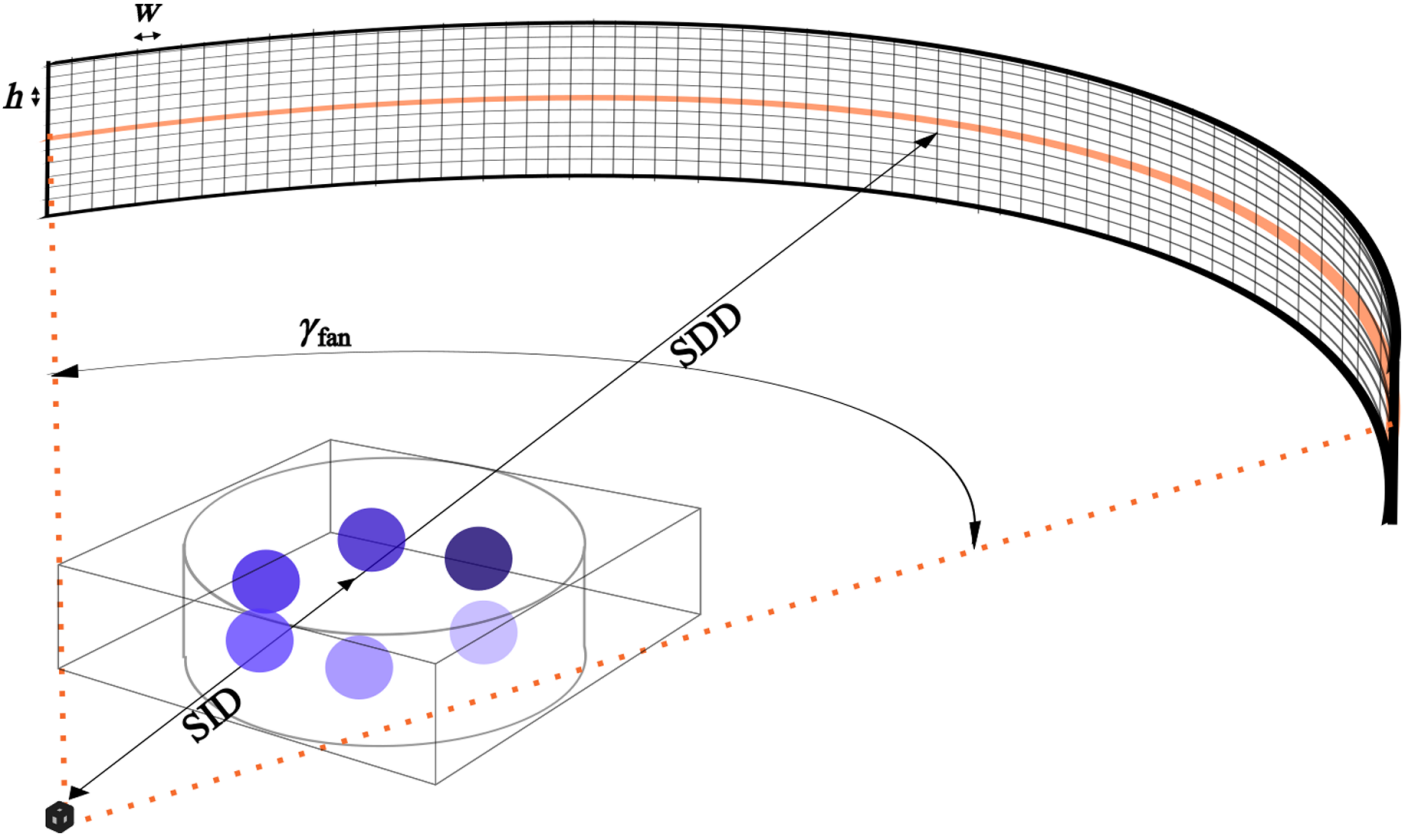
CT geometry used in the present work, with one fan-beam detector slice highlighted. Axial, fan-beam CT simulations were completed for multiple slices of numerical phantoms (not to scale).

The CT scanner geometry was approximately modeled after previous microCT systems.^27^^–^^31^ Geometric parameters included a 100-mm source-to-isocenter distance (SID), 200-mm source-to-detector distance (SDD), 1024 detector channels, and channel detector width (h) equaling the resolution of the discretized phantom. Instead of a flat-panel detector used for some microCT systems, we used a curved detector shown in Fig. 4 (not to scale), with a comparable 19.37 deg fan angle ($\gamma_{\text{fan}}$). We acquired 438 projection views over a 219 deg rotation (greater than $180\;\deg+\gamma_{\text{fan}}$, the minimum rotation required for complete angular data).^32^

We simulated both EICT and PCCT detector schemes. The EICT simulation used an energy-weighted compound Poisson noise model, and the PCCT simulation used a direct Poisson noise model. Perfect counting efficiency was used to provide a matched comparison to XFET simulations. Similarly, the CT source was the complete 120-kVp X-ray spectra that were effectively used for XFET simulations. The CT simulations were highly idealized in that they did not include scatter contamination or electronic noise.

To form an image, we first simulated fan-beam CT images of multiple sequential slices of each discretized phantom, equivalent to a 2-mm slice. We summed photon counts axially over all detector rows to form an image of the 2-mm slice of the object, resulting in images that matched the XFET axial slice width. FFBP with a general sinc window filter was used to reconstruct images with a desired spatial resolution.^32^ A fourth-degree polynomial beam-hardening correction was applied to alleviate cupping artifacts.^32^

### 2.3 Phantom Simulations

The XFET, PCCT, and EICT simulations were performed for the two phantoms described below to compare CNRs and to quantify the detection limit of gold under varied imaging conditions.

#### 2.3.1 MOBY phantom

**Fig. 5:**
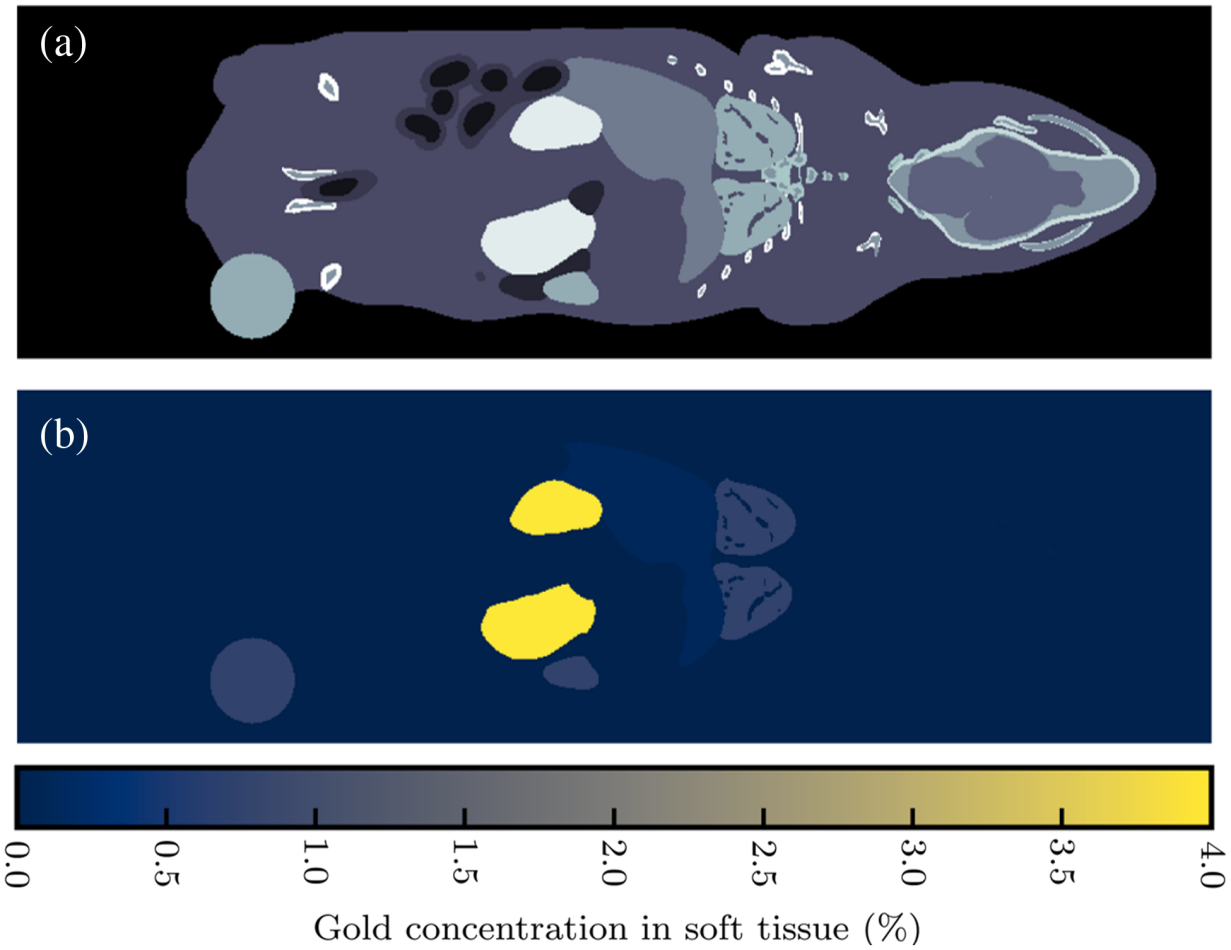
Numerical MOBY mouse phantom. (a) Anatomical map. (b) Map of gold concentrations in specific organs.

The phantom used in this simulation was a discretized numerical mouse phantom, MOBY, displayed in Fig. 5.^24^ This phantom consisted of various International Commission on Radiological Protection (ICRP) tissues, including soft tissue, cortical bone, skeletal muscle, brain tissue, and adipose tissue. Gold nanoparticles at various concentrations were placed in the phantom: 4% by weight in the kidneys; 0.75% by weight in the spleen, lung, heart, and spherical hind leg tumor; and 0.12% by weight in the liver.

XFET imaging of MOBY was designed to test a realistic object in a preclinical setting. XFET simulations were performed as described in Sec. 2.1, with MOBY placed tail first into the pencil beam. MOBY was rastered in 1-mm horizontal and vertical increments through the beam. An equivalent of $1.25\times 10^{8}$ histories was used at each pencil beam position. MOBY XFET images, which were the sum of all detector counts as described above, had 1-mm x- and y-resolutions and a 2-mm axial resolution.

Two 2-mm axial slices of MOBY were scanned with PCCT and EICT: one slice containing the kidneys and another containing the hind leg tumor. CT images were reconstructed with 1-mm x- and y-resolutions to match the XFET resolution. The CT simulations were approximately dose-matched to XFET as both simulations delivered effectively $1.25\times 10^{8}$ histories to a central point with area $(1\;\mathrm{mm}{)}^{2}$.

**Fig. 6:**
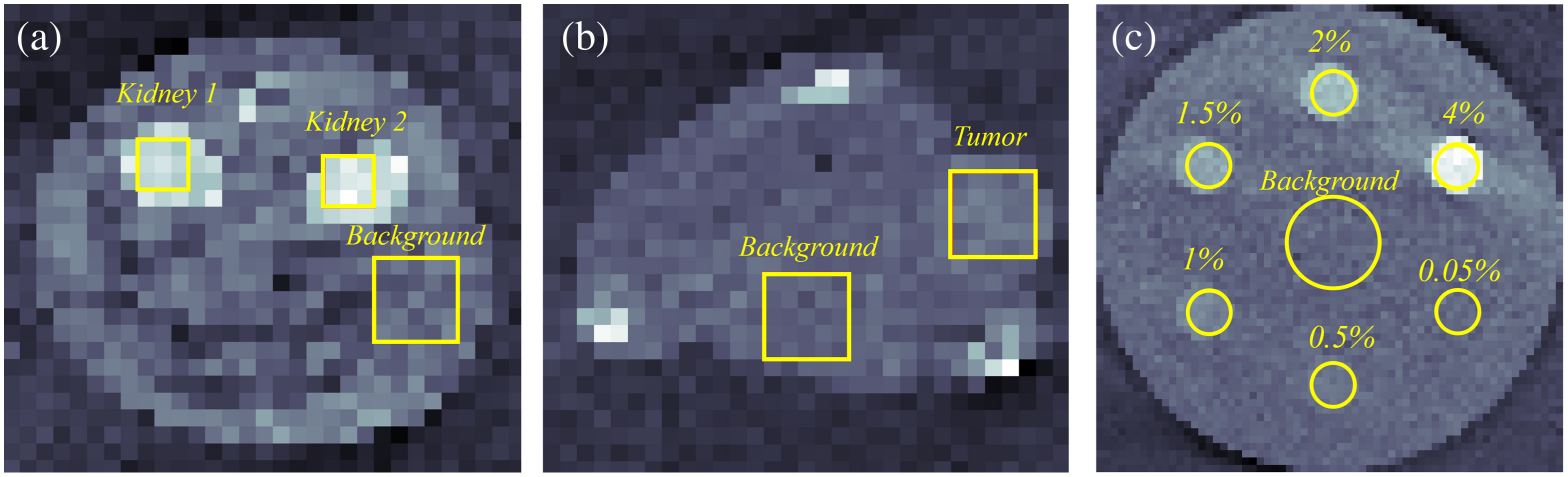
CT images demonstrating ROI placement for the (a) MOBY kidney slice, (b) MOBY tumor slice, and (c) contrast-depth phantom. Within the MOBY phantom, the square ROIs were placed in the background regions, the hind leg tumor, and over each of the two kidneys. In the contrast-depth phantom, the circular ROIs were placed over each gold sphere and in the background region. Displayed ROIs were used for CNR calculations in both XFET and CT images.

In both XFET and CT images, square regions of interest (ROIs) were placed around the kidneys, hind leg tumor, and background abdominal tissue, as shown in Fig. 6. We extracted the average signal from the gold-containing ROIs, $C_{\mathrm{Au}}$, as well as from the background ROIs without gold, $C_{\mathrm{bkg}}$. CNRs were computed for each kidney and the hind leg tumor with

$$\mathrm{CNR}=\frac{C_{\mathrm{Au}}-C_{\mathrm{bkg}}}{\sigma_{\mathrm{bkg}}},$$ (1)

where $\sigma_{\mathrm{bkg}}$ is the standard deviation of the counts in the background ROI. CNRs were compared between EICT, PCCT, and XFET images. The Rose criterion, which defines CNR=4 as the lower limit of signal detection,^33^ was used to quantify the detectability of the organs of interest.

#### 2.3.2 Contrast-depth phantom

**Fig. 7:**
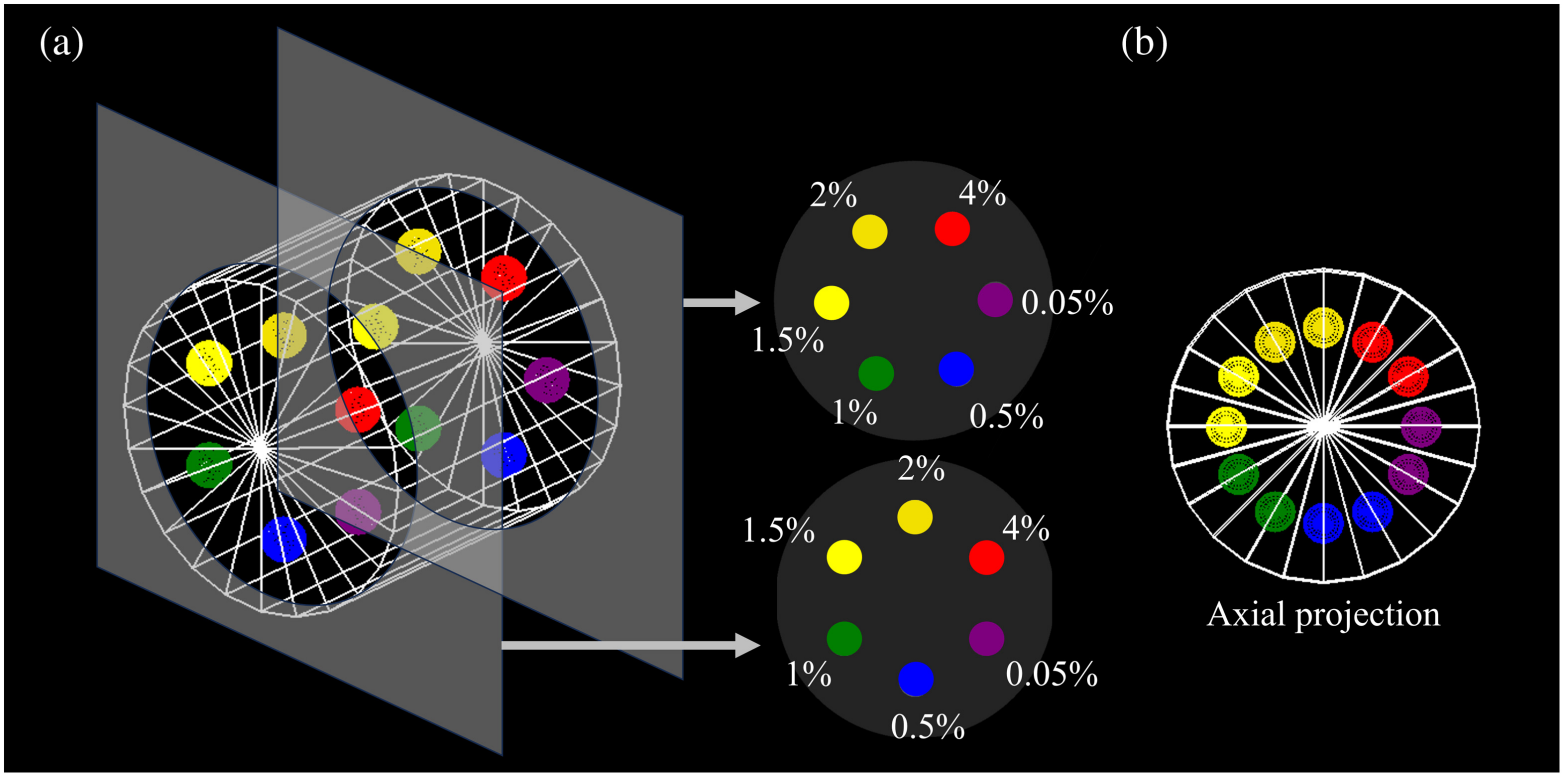
(a) Contrast-depth cylindrical phantom, containing various gold concentrations in soft tissue (shown as % by weight). Although only two depths are shown here for simplicity, four were simulated. (b) Axial projection view of contrast-depth phantom, demonstrating that the gold spheres do not overlap axially.

Although MOBY mimicked a realistic preclinical imaging task, a contrast-depth phantom, shown in Fig. 7, was designed to quantify the conditions under which CT outperforms XFET. Imaging this phantom with both CT and XFET allowed us to study CNR dependence on gold concentration and beam depth.

The cylindrical contrast-depth phantom shown in Fig. 7 was 3.2 cm in diameter and composed of ICRP soft tissue. The phantom contained 4-mm-diameter spherical inserts of 4.0%, 2.0%, 1.5%, 1.0%, 0.5%, and 0.05% gold concentration by weight in ICRP soft tissue. The inserts were placed on two planes of different depths relative to the XFET pencil beam, which traveled parallel to the long axis of the cylinder. This feature tested the effect of beam depth and hardening on XFET contrast performance. A total of four beam depths were tested: 3.25, 28.75, 54.25, and 79.75 mm. The spherical inserts did not axially overlap to ensure the pencil beam was only attenuated by soft tissue before reaching any sphere of interest. The effect of insert-to-object-surface depth on the number of detectable fluorescence photons is known and quantifiable: with knowledge of this depth and the monochromatic fluorescence energy, calculating the degree of attenuation of the signal is straightforward. Thus, insert depth was not studied.

XFET images of the contrast-depth phantom were formed using the geometry and methods described in Sec. 2.1, using an equivalent of $1.25\times 10^{8}$ histories in each pencil beam position and 0.5-mm vertical and horizontal resolutions. A separate simulation was performed to score the total phantom dose using the complete pencil beam spectrum with $1.25\times 10^{8}$ histories.

The CT simulation was performed with the geometry described in Sec. 2.2. In XFET, a pencil beam penetrates along the craniocaudal axis, which reduces the photon flux delivered with increasing distance and results in lower CNR with greater depth. In CT imaging, on the other hand, a fan-beam penetrates perpendicular to this axis, so CNR has no axial depth dependence. For this reason, CT images were simulated for only one sphere-containing plane of the cylindrical phantom.

To image this plane with EICT and PCCT, we considered that each slice of the discretized phantom had a width of 0.0625 mm and aimed for a total slice width of 2 mm to match the axial resolution of XFET images. We simulated fan-beam CT data acquisitions for 32 adjacent slices of the phantom and summed over the detected photon counts for all slices to obtain our final sinogram. CT images were reconstructed to have a matched resolution to XFET: 0.5 mm horizontal and vertical resolutions (corresponding to a 64×64 imaging matrix). We also reconstructed higher resolution images—0.125 mm in both dimensions (corresponding to a 256×256 image matrix)—which were comparable to resolutions produced with microCT.^34^

Both XFET and CT simulations were approximately dose-matched: both used a beam flux intensity of $1.25\times 10^{8}$ photons to a central point of area $(0.5\;\mathrm{mm}{)}^{2}$. More details about dose matching for this phantom and for MOBY can be found in the Appendix.

To compare CNRs produced by each modality, circular ROIs were placed as shown in Fig. 6. Background ROIs were placed in the center of each image and were larger than the gold ROIs to lower the variance of the measurements taken from these ROIs, including the mean and standard deviation of counts.

Five separate images were acquired for XFET, PCCT, and EICT to provide a metric of uncertainty in CNR measurements. This effort included five distinct XFET simulations using different seeds and five separate noise realizations for CT. The number of repeated simulations was chosen on the basis of computational constraints.

#### 2.3.3 Partial FOV imaging

Unlike PCCT and EICT, which require a full sinogram to image any ROI, XFET’s novel detection scheme can perform non-quantitative partial FOV imaging without prior full FOV imaging. We demonstrate this ability here. Note that quantitative imaging requiring attenuation correction might benefit from a full FOV scan to allow for joint estimation of the attenuation map.

After imaging the contrast-depth phantom with the method described in Sec. 2.3.2, we performed an additional, partial FOV, high-resolution XFET scan. The XFET geometry described in Sec. 2.1 remained constant, but this acquisition used a ∼51-fold increase in the local dose ($6.41\times 10^{9}$ histories per beam location) and rastered the pencil beam in 0.25-mm horizontal and vertical increments. The ROI scanned was a ${\left(5.25\;\mathrm{mm}\right)}^{2}$ square centered around the spherical insert containing the lowest concentration of gold (0.05%). This concentration was chosen because it is approximately the theoretical detection limit of CT in typical acquisitions.^8^^,^^35^ Thus, this acquisition aimed to demonstrate not only partial FOV capabilities but also XFET’s detection limit improvement with increased dose.

## 3 Results

### 3.1 MOBY Phantom

**Fig. 8:**
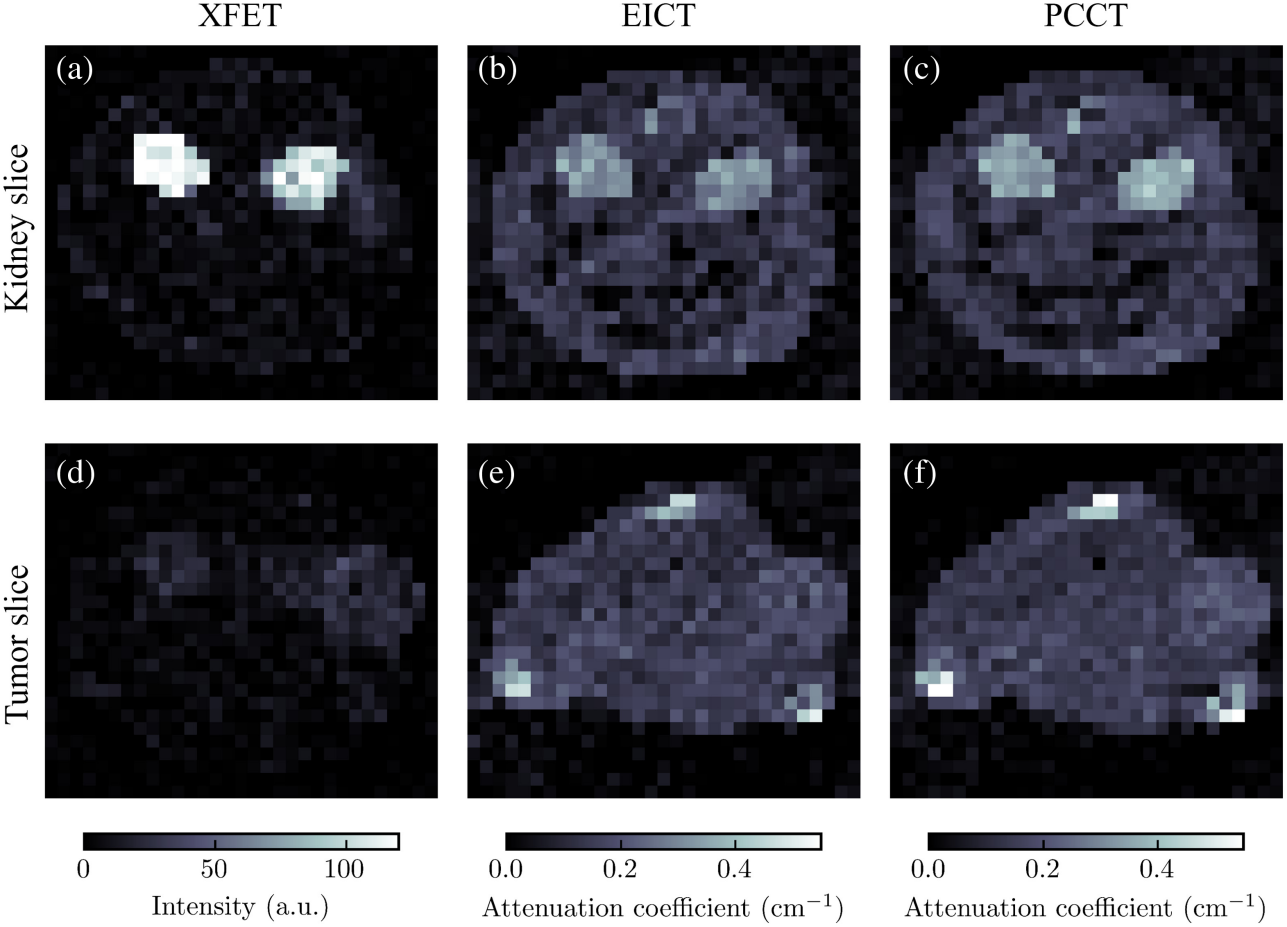
Comparison of axial slices of MOBY imaged with three modalities studied here. The kidneys appear with more contrast in the XFET image than in the EICT and PCCT images. The tumor is less visible due to decreased gold concentration. (a) XFET kidney image. (b) EICT kidney image. (c) PCCT kidney image. (d) XFET tumor image. (e) EICT tumor image. (f) PCCT tumor image.

**Fig. 9:**
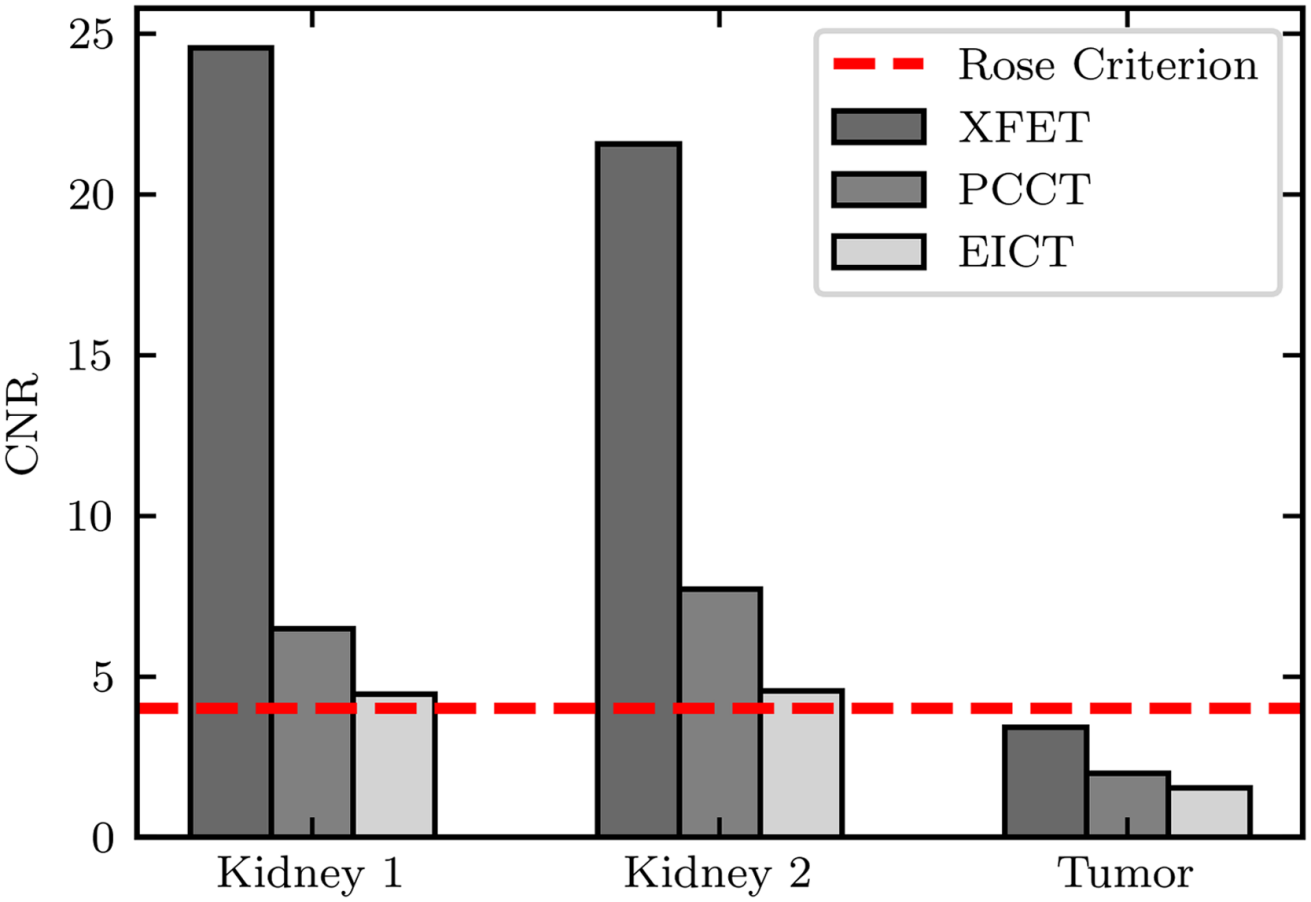
Organ CNRs for each modality. The horizontal dashed line represents the Rose criterion. Bars meeting this threshold indicate organs that would be detectable in imaging.

Figure 8 shows the axial slices of the MOBY phantom imaged with XFET, EICT, and PCCT. The XFET results in greater CNRs for both kidneys and the tumor when compared with either EICT or PCCT. Figure 9 displays a summary of kidney and tumor CNRs; XFET resulted in CNRs of 24.5, 21.6, and 3.4 for the two kidneys and tumor, respectively. Lower CNR values resulted from EICT (CNR=4.4, 4.6, and 1.5) and PCCT (CNR=6.5, 7.7, and 2.0). Every modality could detect both kidneys according to the Rose criterion.^33^ The tumor approached this detection limit when imaged by XFET but fell well below this limit when imaged with EICT and PCCT. Across all three organs of interest, XFET provided an average CNR improvement of 315% compared with EICT and 175% compared with PCCT.

Streak artifacts are present in the XFET coronal view (not shown) and, with close inspection, can be seen in the axial XFET slice containing the tumor as bright “shadows” of the kidneys.

### 3.2 Contrast-Depth Phantom

**Fig. 10:**
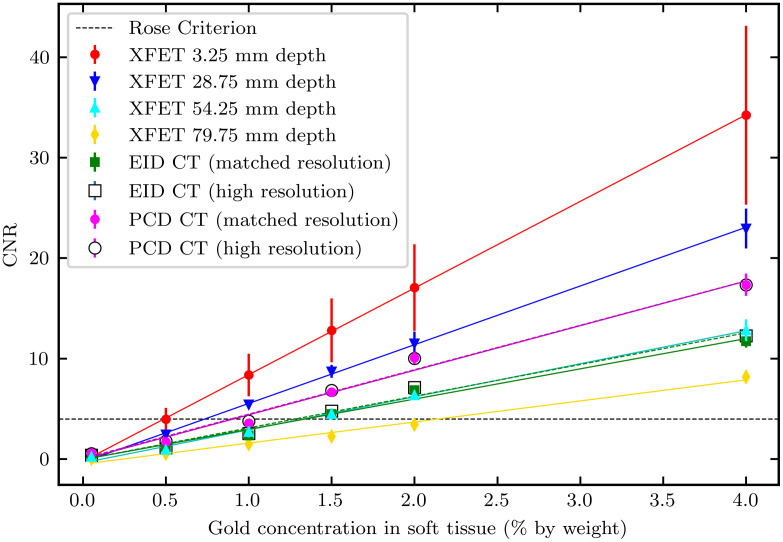
Comparison of CNRs as a function of known gold concentration with XFET and CT in the contrast-depth phantom. Note that the CT results are not depth dependent. At beam depths deeper than 28.75 mm, PCCT outperforms XFET. EICT performs similarly to XFET at a beam depth of 54.25 mm. Error bars indicate the standard deviation of five independent noise realizations.

Figure 10 compares the CNRs obtained from the contrast-depth phantom imaged with XFET, PCCT, and EICT. The 0.5-mm- and 0.125-mm-resolution CT images produced similar results.

As shown in Fig. 10, CNRs are linearly related to known gold concentration in both XFET and CT acquisitions. As expected, CNR increases with increasing gold concentration and decreasing depth of XFET imaging. Superficial XFET (3.25 mm beam depth) produces the greatest CNRs across all gold concentrations. Between the XFET beam depths of 28.75 mm and 54.25 mm, PCCT produces greater CNRs than XFET. EICT produces very similar CNRs to XFET at a 54.25 mm beam depth. Finally, deep XFET (79.75 mm) produced the lowest CNRs across all gold concentrations.

**Fig. 11:**
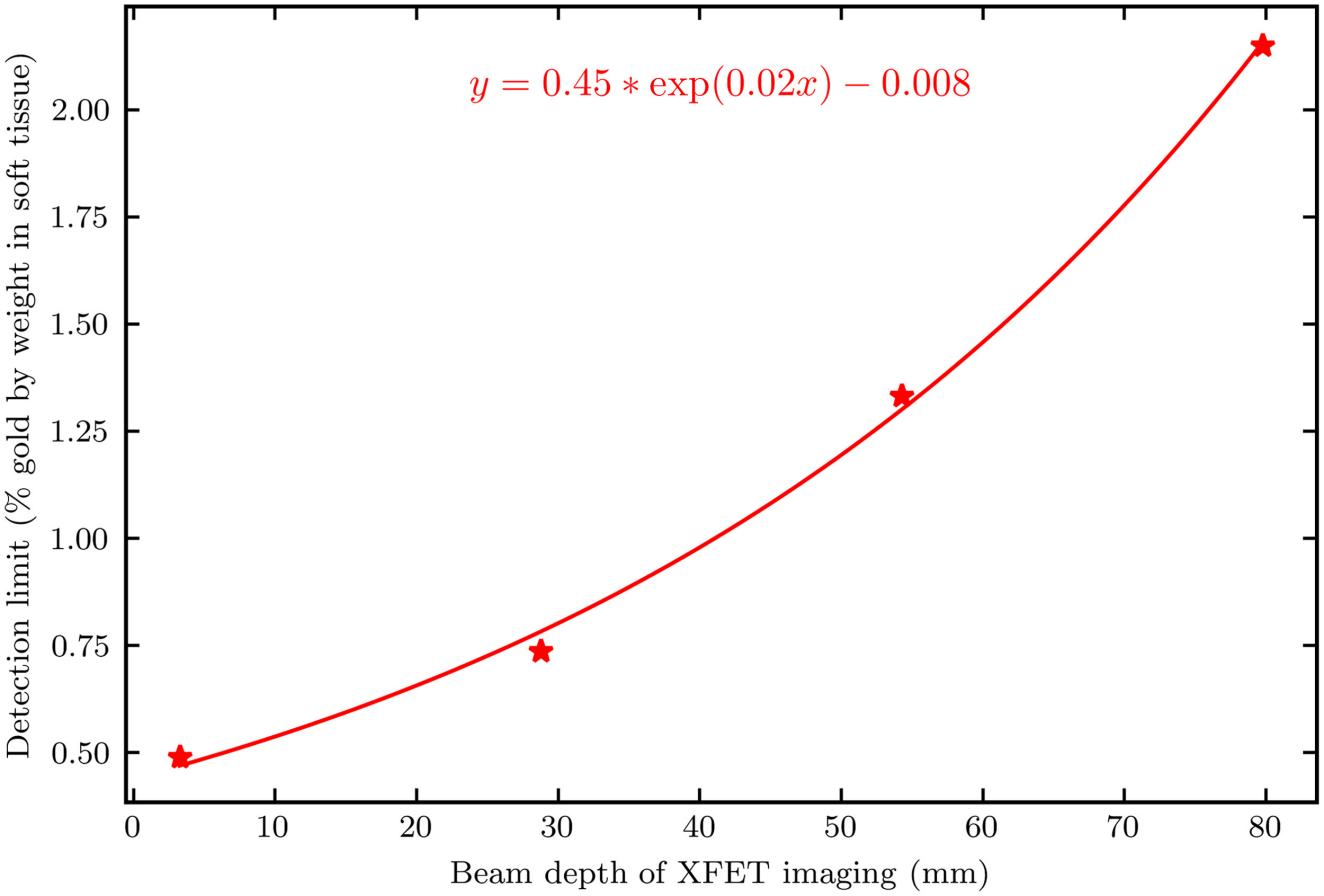
XFET detection limit, as function of imaging depth for 125 million histories (16 mGy).

Figure 10 also contains a horizontal line representing the threshold for the Rose criterion of detectability.^33^ We extracted the intersection between the Rose criterion and XFET’s linear relationship of CNR to gold concentration. These intersections, equivalent to the gold concentration detection limits, are plotted as a function of beam depth in Fig. 11. An exponential fit, given in Fig. 11, was chosen to fit the data because, for a given gold concentration, the primary factor impacting CNR in XFET is beam attenuation. Extrapolating to surface imaging (depth = 0 mm), the XFET detection limit for this phantom dose (16 mGy) is 0.44% gold by weight.

**Fig. 12:**
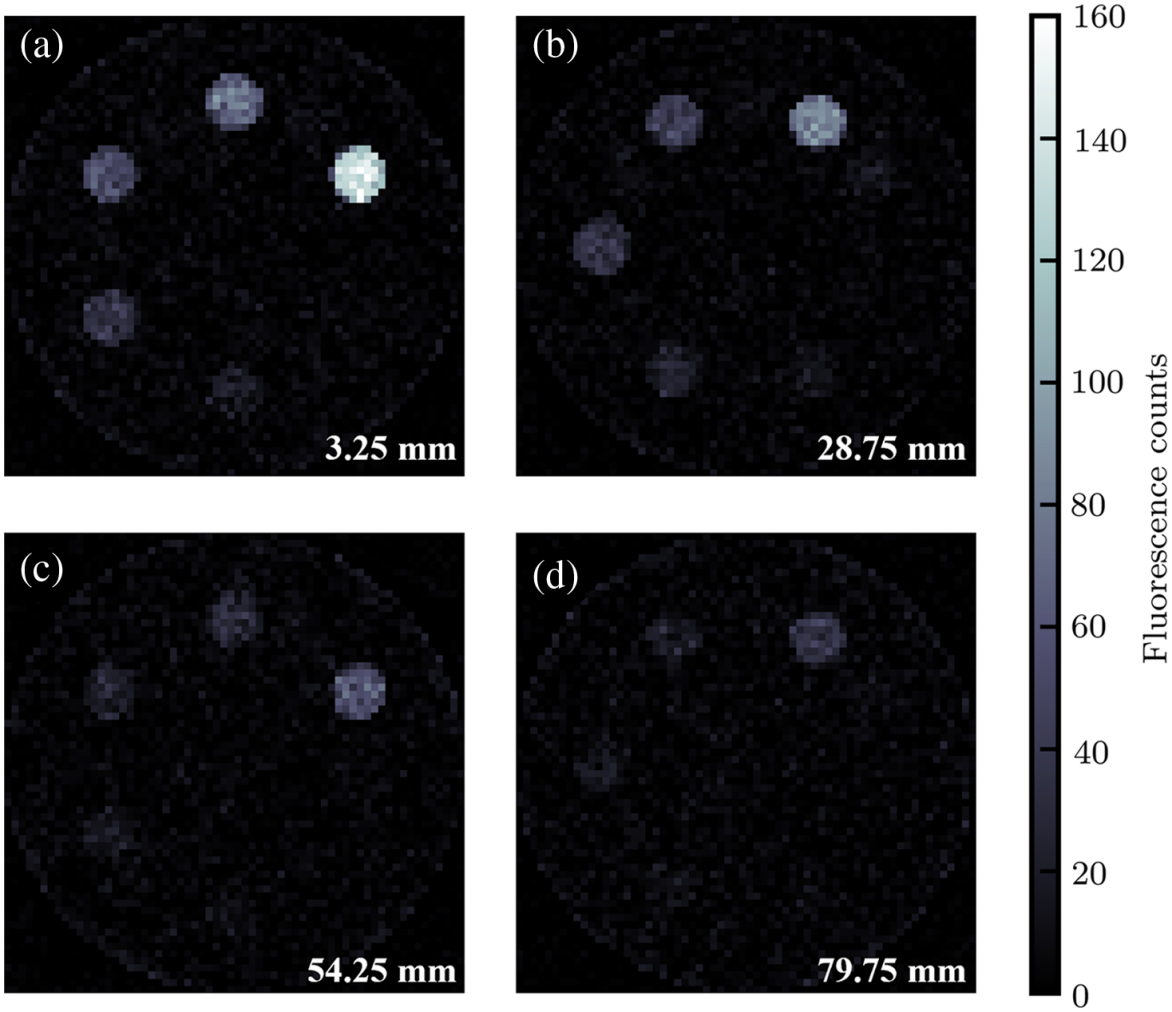
Comparison of XFET images of the contrast-depth phantom at varying depths. (a) XFET at 3.25 mm depth. (b) XFET at 28.75 mm depth. (c) XFET at 54.25 mm depth. (d) XFET at 79.75 mm depth.

**Fig. 13:**
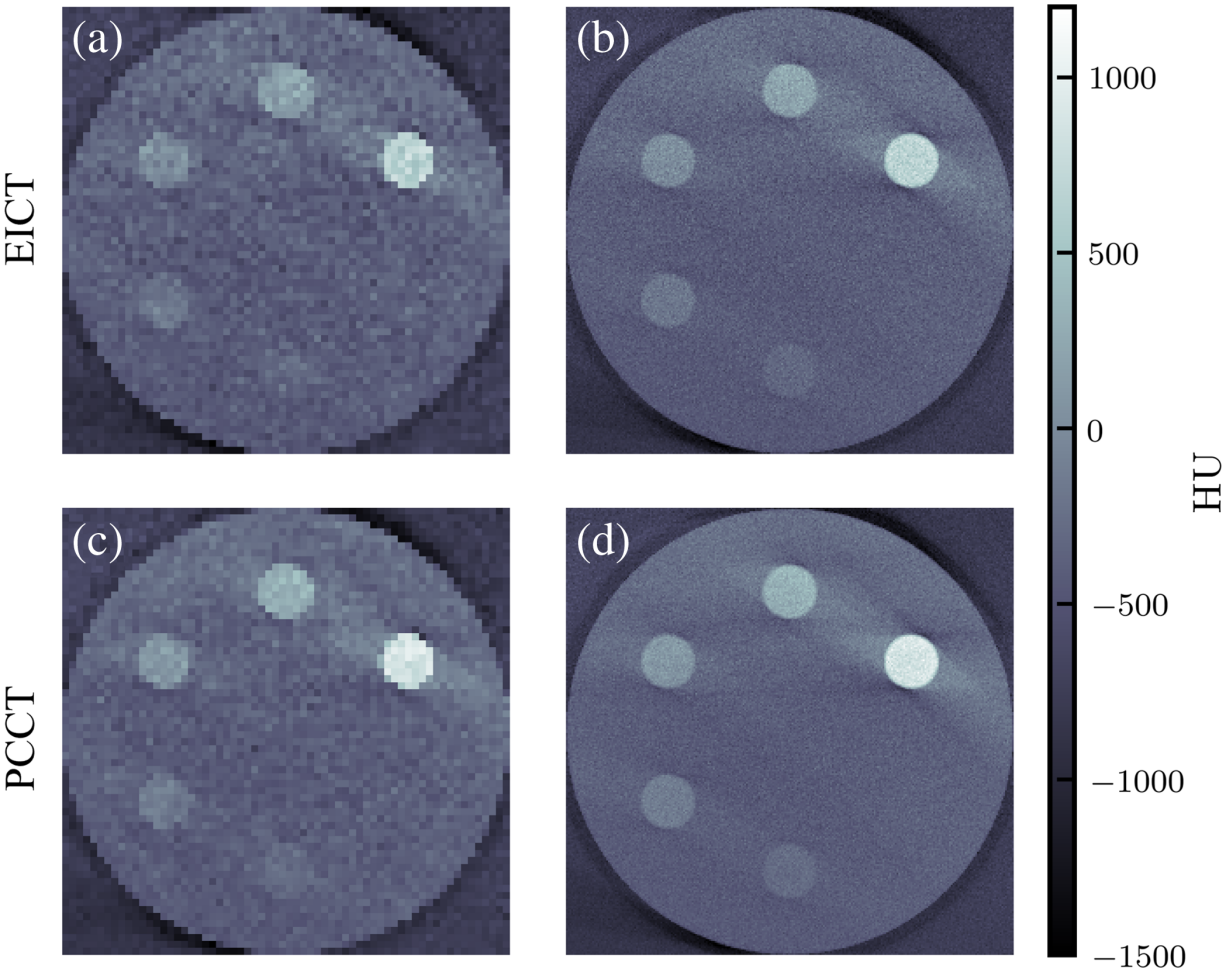
Comparison of EICT and PCCT images of the contrast-depth phantom. (a) EICT images with 64×64 resolution matching XFET. (b) EICT images with 256×256 resolution. (c) PCCT images with 64×64 resolution. (d) PCCT images with 256×256 resolution.

Figures 12 and 13 display the XFET and CT images, respectively, of the contrast-depth phantom. As the XFET imaging depth increases, CNR decreases, and visibility of low concentrations of gold decreases or is lost. CT images, both 0.5-mm- and 0.125-mm-resolution reconstructions, display beam-hardening artifacts, most prominent as streaks between the two highest gold concentrations in each image. CT is able to clearly visualize the background soft tissue cylinder.

### 3.3 Partial FOV Imaging

**Fig. 14:**
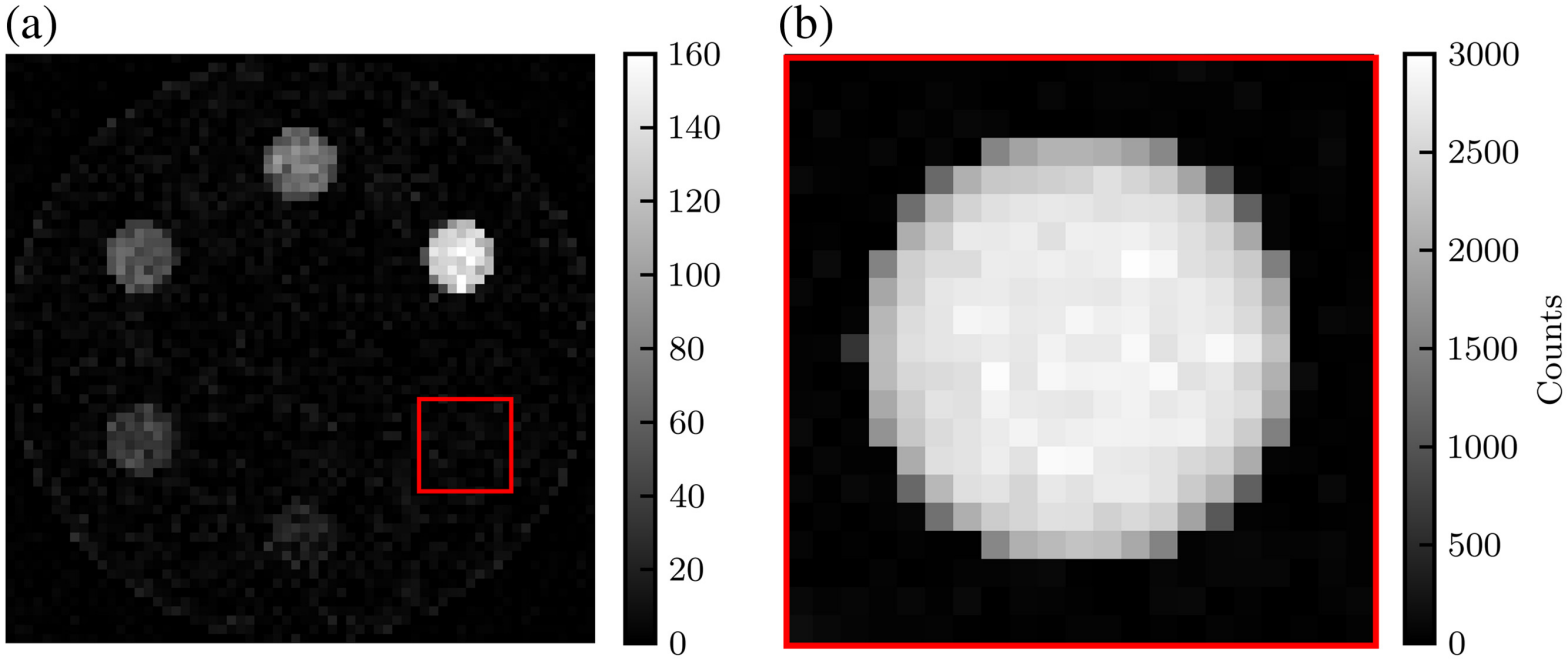
(a) Low-dose XFET image with a square ROI indicating region probed in partial FOV imaging. (b) Partial FOV image of 0.05% gold simulated with high local dose.

Figure 14 displays the full FOV contrast-depth phantom image and the partial FOV image simulated using XFET at a beam depth of 3.25 mm. As demonstrated, the previously undetectable 0.05% gold insert is made visible with a 51-fold increase in imaging dose to this ROI.

## 4 Discussion

XFET is characterized by a novel image acquisition scheme that utilizes pixelated, energy-sensitive, photon-counting detectors in combination with slit apertures and a pencil beam through which a phantom is rastered. Because of this geometry, XFET possesses several advantages over other imaging modalities that give it the potential to be used for metal mapping in certain preclinical applications. As mentioned in Sec. 1, conventional EICT and PCCT that do not utilize spectral information can only distinguish among differences in attenuation. XFET stands apart from these modalities in that it can map many different metals simultaneously by measuring fluorescent energies unique to each metal. Furthermore, XFET is distinct from conventional XFCT in that it is a direct imaging modality that does not measure a sinogram: its mechanism of imaging allows for the possibility of joint estimation and partial FOV imaging. The primary aim of XFET is to provide metal quantification, high sensitivity, and *in vivo* imaging capability for preclinical applications. This work demonstrates XFET’s potential for these tasks. However, certain parameters such as depth and metal concentration must be considered before preclinical translation.

The two modalities studied here, XFET and CT, have different implications for image quality that may affect the metal quantification task. For example, Fig. 13 shows that both EICT and PCCT can suffer from beam-hardening artifacts even with a water-based correction, particularly when imaging greater metal concentrations. Although more advanced beam-hardening corrections can be applied to alleviate such streaking artifacts, these are often bone-based corrections that can require some degree of hand-tuning.^32^ This may have a detrimental effect on metal quantification accuracy. XFET can also suffer from a critical streak artifact due to the combination of X-ray penetration of the lead slit apertures and imperfect background subtraction. This artifact is especially visible for illuminated lines containing high gold concentrations. The artifact only affects the axial direction but can still affect 3D gold quantification, as seen in XFET’s tumor slice in Fig. 8, where a bright “shadow” of the MOBY kidneys could be conflated for gold. Fortunately, this artifact will likely be eliminated with thicker lead apertures and slits with an optimized converging shape, which is implemented for the benchtop XFET hardware.^1^

XFET is also characterized by a resolution–signal tradeoff. In XFET, the axial resolution is determined by the slit width and z-width of each detector pixel. Thus, in this work, the original 1-mm axial resolution was resampled to 2 mm and matched to the CT axial slice width, which worsened the axial resolution but increased the signal and gold visibility. In benchtop preclinical applications, this tradeoff must be considered, and the imaging parameters can be tailored to meet the study requirements. The XFET horizontal and vertical resolutions, however, are determined by beam spread and rastering. While improving the XFET signal through resampling the axial resolution, we maintained high x- and y-resolutions by rastering the phantoms through the beam in small steps, as low as 0.25 mm for the contrast-depth phantom. This characteristic of XFET—the partial independence of axial imaging from horizontal and vertical imaging—is also what allows for partial FOV imaging and probing small regions of interest.

CNRs obtained with XFET are dependent on both beam depth and metal concentration, whereas those obtained with CT are only dependent on metal concentration. Therefore, there is a threshold imaging depth below which XFET outperforms CT. We found that this depth is between 28.75 and 54.25 mm for both PCCT and EICT for the parameters simulated here. Specifically, PCCT outperforms XFET at ∼54.25  mm depth, whereas EICT provides a similar performance to XFET at this depth. At more superficial depths, XFET outperforms both CT detection systems. XFET’s advantage holds for preclinical imaging in which small animal sizes fall below this depth threshold; thus, XFET provides higher contrast for the MOBY tumor and kidneys, as summarized in Fig. 9. For a given superficial depth, it is not surprising that XFET outperforms both EICT and PCCT. Unlike CT, an anatomical imaging modality, XFET is a functional imaging modality: it images metal in regions with a mechanism similar to other emission tomography systems that detect radioactive decay. It is therefore expected that, without beam attenuation limiting the initial pencil beam photon flux, XFET can detect low concentrations of metal with very high contrast.

PCCT unsurprisingly outperforms EICT due to the relative upweighting of lower-energy X-rays. No electronic noise was simulated here, but in real detection systems, EICT will suffer from electronic noise effects, whereas the PCCT detection scheme will not. This difference will increase the disparity of contrast performance between the two systems. Spectral photon-counting CT (SPCCT) utilizes spectral information to perform material differentiation and K-edge imaging.^36^ However, SPCCT is usually characterized by a limited number of spectral bins^37^ and utilizes CdTe or silicon detectors with energy resolutions ranging from 3.5 to 10 keV.^38^ By contrast, the CdTe HEXITEC detectors used in XFET are characterized by a high spectral resolution (1 keV) that is required for fluorescence imaging.^2^ This energy resolution, in combination with XFET’s direct detection of fluorescence without image reconstruction, allows for the differentiation among many different metals with no theoretical loss of CNR. XFET is also characterized by a low fluorescence emission rate and thus a low photon detection rate, which makes XFET imaging slow, but eliminates the detrimental effects of pulse pile-up that reduces signal, reduces dose efficiency, and distorts material differentiation in photon-counting CT.^38^^–^^40^ This combination of XFET’s detection characteristics—including its high spectral resolution and direct image acquisition scheme—gives XFET the potential for improved metal imaging relative to PCCT.

For all imaging modalities, the relationships between CNR and gold concentration are linear and do not overlap, indicating that, for a fixed depth, the order of modality performance was not dependent on gold concentration. This linearity was expected due to the linear relationship between gold concentration and fluorescence emission in XFET and the approximate linear relationship between gold concentration and attenuation coefficient in CT. This linearity also indicates that XFET is capable of quantitative metal imaging even without image reconstruction for simple objects. However, image reconstruction for attenuation correction may prove useful in realistic metal quantification tasks in which gold regions are at varying radial depths or the object has spatially varying attenuation. The image reconstruction algorithm that we previously developed will be implemented in future work to address this need.^16^ We expect this image reconstruction to improve XFET’s image uniformity and reduce the contrast dependence on depth.

XFET signal is also dose-dependent: for a N-factor dose increase, one expects the CNR to increase by a factor of $\sqrt{N}$. For ideal CT systems neglecting pulse pile-up effects, such as the one modeled in this work, we expect the relationship between CT and XFET CNRs to be preserved with dose because both CNRs would increase by the same factor for both modalities. However, pulse pile-up reduces dose efficiency in PCCT, and realistic PCCT CNR would increase at a slower rate depending on the count rate capabilities of the detector at high fluence rates.^38^ Because XFET is characterized by a low photon detection rate, it does not suffer the effects of pulse pile-up, and therefore, CNR is expected to increase as expected with dose. This dose dependence is demonstrated in this work: the 0.05% gold sphere was originally not visible in Fig. 12, which used $1.25\times 10^{8}$ histories, but became very visible in the partial FOV image (Fig. 14), which used a 51-fold increase in local dose (16  mGy×51=81.6  cGy). This demonstration also highlighted a useful function unique to XFET but not CT or conventional XFCT: XFET allows for whole FOV imaging followed by partial FOV imaging for an ROI, with excess dose only delivered to the ROI. This capability follows from XFET’s direct imaging of a non-rotating object without sinogram acquisition. Imaging with a similar sequence would be especially beneficial for examining organs of interest in preclinical imaging. Although benchtop XFET may take on the order of an hour to acquire a full image,^1^ slow imaging times can be improved over conventional XFCT when utilizing this partial FOV capability.

Aside from the partial FOV image, most results of this work are specific to the relatively low dose used in this study. This total dose to the contrast-depth phantom was scored by TOPAS as ∼16  mGy and therefore is conducive to *in vivo* imaging. For this dose, the XFET detection limit varies with beam depth according to the exponential equation in Fig. 11. For surface imaging (depth=0  mm), the detection limit would be ∼0.44% gold by weight. Taking dose into account by finding the dose-detection limit product (7.04%-mGy), the XFET sensitivity reported here outperforms conventional XFCT according to previous reports^21^^,^^41^ and performs similarly to XFCT with optimized backscatter or full-ring detector geometry.^22^^,^^42^ XFET’s detection limit can be further improved using a filtered or monochromatic beam, optimizing slit hardware, or increasing the dose, especially to small ROIs.

The detection limit and dose reported here are sufficient for some preclinical imaging tasks, such as guiding metal-mediated radiation therapy.^10^^,^^43^ Because XFET outperforms CT for superficial imaging, XFET would be preferable for mapping metal nanoparticles used in the treatment of skin disease or superficial tumors.^44^^–^^46^ Its partial FOV imaging capability allows for *in vivo* biodistribution imaging of metal nanoparticles for the development of metal-based drugs. However, there are some limitations of this work that warrant further study before XFET’s sensitivity and detection limits are finalized.

One limitation of this study is the use of 100% detector counting efficiency. Perfect photon counting was chosen to obtain a standardized comparison of CT and XFET. Real detectors would likely result in slightly lower CNRs for both modalities, but this CNR decrease could be further overcome in XFET with hardware optimization or utilization of higher dose partial FOV imaging. CdTe detectors used in CT imaging may suffer from reduced counting efficiency due to pulse pile-up effects. XFET, on the other hand, is characterized by a low fluorescence detection rate, and therefore, its detectors would not experience detrimental pulse pile-up. Furthermore, the 1-mm-thick CdTe HEXITEC detectors are expected to absorb 96% of gold’s fluorescence emissions at ∼69  keV: similar to the perfect counting efficiency modeled here. We also did not model charge sharing or interpixel cross-talk in these ideal detector models. Because our benchtop XFET system has active pixel sizes similar to previously modeled micro-CT systems,^1^^,^^28^ we expect that, if these effects were modeled, XFET and PCCT would display similar rates of image quality degradation due to these effects. Thus, the modeling of these effects will be considered in future investigations that study the absolute, not relative, performance of these modalities. Second, this study did not incorporate scatter in CT simulations, resulting in highly idealized CT images. Scatter contamination of real data would result in lower CT CNR, so XFET is likely to outperform CT at even greater depths than shown here. Metrics for CNR that incorporate ROI variance may be less favorable to XFET, which may balance XFET’s advantage. However, the CNR definition used here is a more commonly reported metric in the literature and is often used when determining detection limits based on the Rose criterion.^1^^,^^22^ We also expect CNR results to depend on the insert size, which was not studied here: large inserts could potentially self-attenuate in XFET.^22^ The 4-mm-diameter gold spherical inserts were chosen to be similar in size to the kidneys and tumor of the MOBY phantom, that is, realistically sized ROIs for preclinical imaging. Finally, we did not use spectral information offered by SPCCT to perform material decomposition in this work. Similar to XFET, SPCCT can distinguish among many different metals, but it has been shown that its detection limit for gold does not vary significantly from conventional CT.^47^ With the scope of this work encompassing detection limits and sensitivity differences among modalities, the use of non-weighted SPCCT may not offer additional relevant information beyond the PCCT detection system presented here. Regardless, modeling and comparing SPCCT will be performed in future work.

The performance differences between CT and XFET may also depend on source specifications such as energy spectrum, maximum kVp, and filtering. For example, monoenergetic beams above gold’s K-edge are much more dose-efficient in XFET imaging because every incident photon has the potential to induce fluorescence emissions. Understanding the effect of source specification on performance differences among the modalities is an area of future investigation.

Before XFET can be translated to preclinical work, the metal of interest must be considered. Different atomic number metals have varying photoelectric cross sections, fluorescent yields, and energy of X-ray fluorescent emissions that may impact the contrast seen in XFET as well as CT, which will produce varying results. For example, iodine is sometimes difficult to detect with spectral PCCT due to low numbers of photons around its K-edge energy (33.2 keV),^36^^,^^47^^,^^48^ whereas fluorescent photons around this energy are easily induced and measured with XFET.^1^ Although gold was used here due to its significance in novel therapies, our group has also shown that XFET using a multi-pinhole aperture and HEXITEC detectors has resulted in significantly improved detection limits for Gd (0.01%) compared with studies that utilize a similar dose (∼3.26  Gy).^1^ Exploring other metals is another area of future direction and a critical investigation before preclinical translation.

## 5 Conclusion

This work provided the first comparison of XFET and CT for detecting low concentrations of gold in soft tissue and characterized the conditions under which XFET outperformed EICT and PCCT. Using both Monte Carlo XFET and analytical fan-beam CT simulations to image a realistic whole-body mouse phantom, we showed that XFET provided greater CNRs than CT for 4% gold (Au) in the kidneys and 0.75% Au in a hind leg tumor. Performing these simulations with a contrast-depth phantom, we showed that XFET outperformed CT for superficial imaging depths (<28.75  mm) for gold concentrations above 0.5%. XFET’s surface detection limit was quantified as 0.44% for an average phantom dose of 16 mGy, which is compatible with *in vivo* imaging. Finally, XFET’s ability to image partial FOVs was demonstrated, and 0.05% Au was easily detected with an estimated dose of ∼81.6  cGy to the small ROI. Thus, this work demonstrated a proof of XFET’s benefit for imaging low concentrations of gold at superficial depths in preclinical imaging tasks.

## 6 Appendix: XFET and CT Dose Matching

In the XFET simulations, a fixed number of photons, $I_{b}$, was delivered in each beam location. The cross-sectional area of each beam location, $A_{0}$, was determined by the horizontal and vertical resolution targets, with $A_{0}$ equal to ${\left(0.5\;\mathrm{mm}\right)}^{2}$ for the contrast-depth phantom and ${\left(1\;\mathrm{mm}\right)}^{2}$ for MOBY. The larger area for MOBY was chosen due to the computational constraints of voxelized phantoms. To approximately dose match the CT simulations, we aimed to deliver $I_{b}$ photons to an area $A_{0}$ of the object at the source-to-isocenter distance (SID) over the course of CT imaging. Some terms below can be seen in Fig. 4, which displays a schematic of CT imaging.

Let N be the target total counts per area $A_{0}$ over all CT projection angles, given as

$$N=\frac{I_{b}}{A_{0}}.$$ (2)

In our fan-beam CT simulations, the height of a detector element, h, was set equal to the width of one z-slice of the phantom. The width of one detector element, w, can be projected onto the isocenter, where it will have width $w_{\mathrm{iso}}$.

The arc length of the complete fan beam at the SDD, $w_{\text{fan}}$, is given as

$$w_{\text{fan}}=\gamma_{\text{fan}}\mathrm{SDD},$$ (3)

where $\gamma_{\text{fan}}$ is the fan angle. The width of one detector element is $w_{\text{fan}}$ divided by the number of detector channels, $N_{\text{channels}}$, given as

$$w=\frac{w_{\text{fan}}}{N_{\text{channels}}}=\frac{\gamma_{\text{fan}}\mathrm{SDD}}{N_{\text{channels}}}.$$ (4)

The value of $w_{\mathrm{iso}}$ the given using Eq. (4) and rules of similar triangles as

$$w_{\mathrm{iso}}=w\frac{\mathrm{SID}}{\mathrm{SDD}}\;=\frac{\gamma_{\text{fan}}\mathrm{SDD}}{N_{\text{channels}}}\frac{\mathrm{SID}}{\mathrm{SDD}}\;=\frac{\gamma_{\text{fan}}\mathrm{SID}}{N_{\text{channels}}}.$$ (5)

Finally, we calculate how many photons need to be delivered to one detector channel for one projection angle: $I_{\mathrm{proj}}$. This is the target counts per area (N), multiplied by the area of one detector element projected onto the isocenter $\left(h\times w_{\mathrm{iso}}\right)$ and divided by the number of projection angles $N_{\mathrm{proj}}$, given as

$$I_{\mathrm{proj}}=\frac{N}{N_{\mathrm{proj}}}(h\times w_{\mathrm{iso}}).$$ (6)

Thus, to approximately dose match our CT simulations to XFET, we used $I_{\mathrm{proj}}$ number of photons delivered to each detector channel for each projection angle. XFET simulations used an effective $I_{b}=1.25\times 10^{8}$; therefore, CT simulations used $I_{\mathrm{proj}}=2356$ for the contrast-depth phantom and $I_{\mathrm{proj}}=1177$ for the MOBY phantom (the difference results from the differences in phantom resolution and target XFET resolution).

## Data Availability

The code used to generate and analyze the data in this paper can be freely accessed through GitHub at https://github.com/hadleysmith/xfet-mc for the XFET simulations and analysis and https://github.com/gjadick/xtomosim for the CT simulations.
